# Has Latin America achieved universal health coverage yet? Lessons from four countries

**DOI:** 10.1186/s13690-022-00793-7

**Published:** 2022-01-21

**Authors:** Ramiro E. Gilardino, Pilar Valanzasca, Susan B. Rifkin

**Affiliations:** 1 Health Services Research, Distance Learning Program. The London School of Hygiene and Tropical Medicine, University of London, London, UK; 2 Independent Researcher and Healthcare System Consultant, Miami, FL USA

**Keywords:** Universal health coverage, Latin America, Health systems, Healthcare access, Primary healthcare, Healthcare financing

## Abstract

**Background:**

Seven years after the commitment to United Nations’ call for Universal Health Coverage, healthcare services in Argentina, Brazil, Colombia, Mexico are generally accessible and affordable; but they still struggle to meet population health demands and address the rising health care costs. We aim to describe measures taken by these four countries to commit by Universal Health Coverage, addressing their barriers and challenges.

**Methods:**

Scoping literature review, supplemented with targeted stakeholders survey.

**Results:**

The four countries analysed achieved an overall index of essential coverage of 76–77%, and households out of pocket health expenditures fall below 25%. Services coverage was improved by expanding access to primary healthcare systems and coverage for non-communicable diseases, while provided community outreach by the increase in the number of skilled healthcare workers. New pharmaceutical support programs provided access to treatments for chronic conditions at zero cost, while high-costs drugs and cancer treatments were partially guaranteed. However, the countries lack with effective financial protection mechanisms, that continue to increase out of pocket expenditure as noted by lowest financial protection scores, and lack of effective financial mechanisms besides cash transfers.

**Conclusions:**

Argentina, Brazil, Colombia, and Mexico have made progress towards UHC. Although, better financial protection is urgently required.

**Supplementary Information:**

The online version contains supplementary material available at 10.1186/s13690-022-00793-7.

## Highlights

- This review presents the current situation of UHC implementation in Argentina, Brazil, Colombia, and Mexico, using different elements from the WHO in their 13th Program of Work to compare service coverage and financial protection.
- During the 1990s, health systems within several Latin American countries, anticipating to Universal Health Coverage increasing the service coverage, but struggling to fulfill financial protection standards. Still the four countries struggle to find mechanisms that could increase pooling mechanisms capable of increasing service coverage, while reducing financial inequities among people.
- The decentralization of the primary healthcare system, the development of public-private partnerships, and the implementation of progressive financing mechanisms like conditional cash transfers are potential manners to improve service delivery and financial protection contributing to effective UHC.

**Supplementary Information:**

The online version contains supplementary material available at 10.1186/s13690-022-00793-7.

## Background

According to the World Health Organization (WHO), comprehensive universal health coverage (UHC) strategies aim to guarantee “universal access to a strong and resilient people-centered health system, with primary care as its foundation” [1]. Currently, 1.3 billion people lack access to effective, affordable healthcare, while an additional 1.7 billion spend at least 40% of their household income on healthcare [2].

In their 13th General Program of Work (GPW), the WHO seeks to expand UHC to one billion additional people by 2030, benefiting from the Sustainable Development Goals (SDGs) 3. Despite that WHO Regional Offices implemented roadmaps to track advances towards UHC including several domains, the 13th GPW provides an updated framework to support country level strategies to achieve both service coverage and financial protection, there are six areas included in this new framework: Services Access and Quality; Healthcare Workforce; Access to Medicines, Vaccines, and Health Products; Governance and Finance; Health Information Systems, and Advocacy [3–7].

The countries must account to progressively increase public healthcare financing and access to essential healthcare services, covering prevention, health promotion, treatment, and rehabilitation, especially to vulnerable groups [8]. UHC must also include financial protection initiatives avoiding the potentially catastrophic impact of large medical bills [7]. The WHO notes that policies that improve access to health services have had greater impact on expanding UHC than those that improve financial protection [9].

During the 1990s, several countries throughout Latin America (LAC) began reforming their healthcare systems by creating frameworks to monitor improvements in quality of care [3], enhancing primary healthcare (PHC) networks [10], decentralizing health governance, strengthening regulatory measures, and improving efficiency [7]. They also addressed the structural fragmentation that prevented health providers from making purchasing decisions. Yet these efforts have been challenged by inequitable funding and employment-based contributions that sometimes create parallel payment schemes that can lead to tiered and fragmented care [11]. As countries allocate financial resources differently, many developing economies are still debating which UHC financing mechanism may best serve their country [12].

The purpose of this paper is to examine the approaches, challenges, and barriers to implement UHC in four countries of LAC (Argentina, Brazil, Colombia, Mexico).

As summarized in Table 1, these four countries have all implemented UHC with each country achieving an overall index of essential coverage of 76–77%. And each of these counties has expanded financial protection for their citizens with less than 5% of their population incurring health expenditures greater than 25% of their total household expenditures. But as demand for health services increase, each country now struggles to ensure access to and affordability of health services. Differences in how these countries implemented UHC – how services are provided and funded - now impact their ongoing struggles to meet their growing health care needs. Understanding similarities and differences in how these countries implement UHC is critical to helping other LACs develop health policies that can best support their citizens. This paper concludes by making recommendations to help other LAC strengthen their health systems to fully commit to UHC.

**Table 1:** Overview of study countries (population, UHC implementation)

	Argentina	Brazil	Colombia	Mexico
Population (2019) *million*	44,9	211.0	50,3	127,6
GDP x capita (2019) *USD*	10,006.1	8717.2	6432.4	9863.1
Health Expenditure *%GDP*	9.12%	9.47%	7.23%	5.52%
UHC Implemented	Yes	Yes	Yes	Yes
Overall index of essential coverage	76%	77%	77%	76%
Financial Protection ^i^	4.7%	3.46%	2.23%	0.23%

i = % of citizens with health expenditures greater than 25% of total household expenditures

## Methods

We conducted a scoping literature review from January 2012 through January 2019 in Pubmed and Lilacs (See search strategy presented in Appendix 1). We also performed a grey literature search (policy reviews, white papers, Ministries of Health (MOH) web pages, global and regional organization web pages). Timeframe was selected from the year prior to 7 year after the United Nations General Assembly Resolution 44/225, in which the four countries agreed to commit with UHC. Two reviewers (REG, PV) screened each publication’s title and abstract without language distinction (Spanish, English and Portuguese). Articles that included UHC components related to health services coverage or financial protection underwent full text assessment by REG. Discrepancies over whether to include specific articles in the review were resolved through team consensus.

In addition, we surveyed three regional stakeholders to corroborate published information, address evidence gaps, and support the policy recommendations development.

### Evidence assessment

Full text assessment and data extraction was guided by an evidence matrix created using three core categories represented in the WHO 13th GPW framework as well as UHC Regional Roadmaps: i) Health service delivery: analyzed the strengthening the provision of healthcare including PHC; ii) Access to medicine and health products: explored strategies for granting timely and quality access to medicines, vaccines, medical technologies access; iii) Financing, governance, stewardship and health information systems: convened actions to promote adequate health financing, like development of mechanisms of revenue generation, resource pooling and strategic purchasing; and improvement in health information systems capable to monitor health determinants and provide adequate health statistics.

**Fig. 1:**
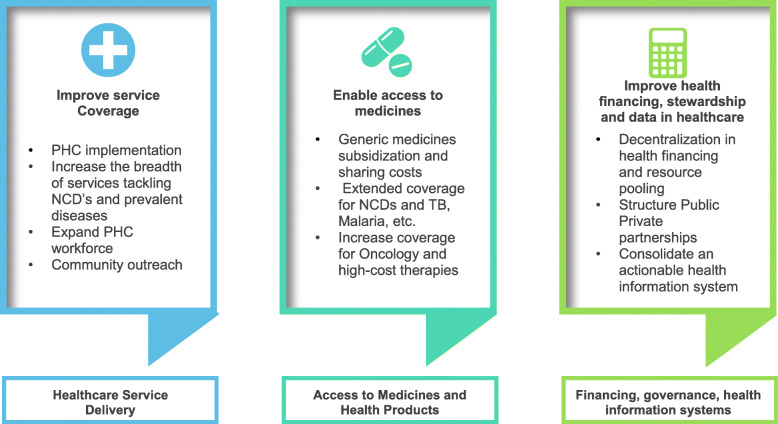
Graphic representation of the evidence assessment matrix. Source: Own elaboration with adaptations from regional frameworks for UHC assessment and the WHO 13^th^ GPW

Figure 1 represent the elements employed to appraise the evidence.

## Results

**Fig. 2:**
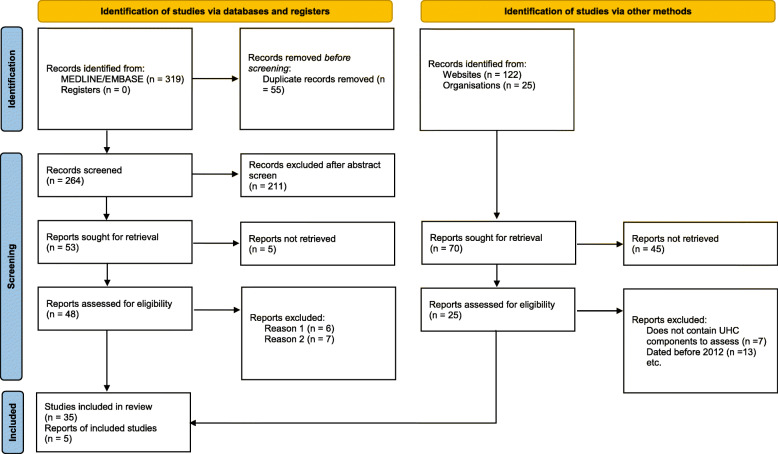
Flow Diagram of Literature search process. *Modified from* Page MJ, McKenzie JE, Bossuyt PM, Boutron I, Hoffmann TC, Mulrow CD, et al. The PRISMA 2020 statement: an updated guideline for reporting systematic reviews. BMJ 2021;372:n71. doi: 10.1136/bmj.n71

Abstracts from 411 peer-reviewed and grey literature articles were screened, with 73 peer-reviewed underwent full-text assessment. A total of 40 peer-reviewed articles were selected for qualitative synthesis. Of these 40 articles, four focused solely on Argentina, 14 on Brazil, one on Colombia, and 13 on Mexico. The remaining eight articles examined health system processes in multiple countries (one article focused on Argentina, Brazil and Mexico; one on Brazil and Mexico; and three examined Colombia and Mexico). The three final articles reviewed processes from across Latin America. Figure 2 includes a modify PRISMA® chart that includes the study selection process.

We present a narrative review identifying achievements and challenges faced by the four countries during UHC implementation. In the Appendix 2 (supplementary material), a detailed comparison between countries is provided.

### Healthcare service delivery

Nearly all articles described strengthening delivery of primary care services, either by creating new care delivery models or by enhancing already functioning services [13, 14]. The evidence suggests that some approaches reduced health inequities [15–18]. Table 2 summarizes the findings from the countries healthcare systems.

**Table 2:** Overview of country level healthcare systems

	Argentina	Brazil	Colombia	Mexico
UHC Commitment	Yes	Yes	Yes	Yes
Healthcare systems Characteristics and Organization	Tripartite system (Public, Social Security and Private health plans) (All citizens are entitled to use the public sector despite having other forms of coverage.)	Dual system: Public Sector (“Sistema Único de Saúde - SUS”) and Private (Supplemental Health (SHS))	MoH through *Plan Obligatorio de Salud* (POS) [Mandatory Health Plan] determine the health coverage. Private health plans and Armed Forces plans also exist for certain subgroup	Tripartite system: Public sector, Social security (IMSS, ISSTE, PEMEX covers more than 70% of population) and private insurance.
Healthcare services Provision	Public sector manages their healthcare facilities at each level. Social security and Private sector provide through its own healthcare facilities or contracting other private providers.	Healthcare facilities are managed at municipal and state level through hospitals and primary care centers. Private sector is contracted by the MoH to provide advanced care or reduce waiting lists.	Private organizations (EPS – Health Providers Organizations) administrate and deliver care either own (IPS) or contract private institutions to provide healthcare. EPS also provides private health plan with copayments.	Social security institutions own their healthcare facilities where provide services. Public hospitals cover “Seguro popular” or the uninsured. Private facilities also provide services through arrangements with social security or by fee for services.
Public Healthcare Financing Mechanisms	General Taxes +Provincial and Municipal Taxes for each sublevel.	General Taxes + Federal, Statal and municipal budgets.	Districts or municipal taxes + Government contributions and central taxes.	General and State Taxes. The Central Governments subsidize 50% of “*Seguro Popular*”.

*The Argentinean Plan Federal de Salud* [Federal Health Plan], a national PHC program including pharmaceutical policies, maternal and infant health and public health insurance was created in 2004 later in 2007 the *Plan Nacer* [Plan to Born] was introduced, providing coverage to pregnant women and children up to 5 years of age [19]. In 2013, was renamed *Plan Sumar* [Plan To Add] expanding coverage to at-risk and low-income citizens. Since its inception, the low birth weight mortality rates have dropped 9%, while neonatal mortality has fallen 22% [18, 20]. Also, the number of deliveries attended by skilled healthcare workers increased among women living below the poverty level [15, 16, 18, 21].

Brazil’s PHC network provide coverage to 62% of the population. The introduction of *Estrategia de Saúde Familiar* [Family Health Strategy] (ESF), a community-based multidisciplinary program that assists vulnerable populations by engaging community healthcare workers at the PHC level*,* reduced health inequalities among racial groups and achieved higher quality of care and user satisfaction compared to PHC or private healthcare [10, 15, 17, 22].

In Colombia Law 100 (1993) established the creation of *Plan Obligatorio de Salud*,*[mandatory health plan – POS]* covering the entire population by two regimens, the contributory regimen for those under formal employment and the Subsidized for the unemployed. This model allowed the managed competition through large-scale participation of the private sector, while also separating the purchasing functions from care provision [22].

Finally, in 2004, Mexico established the health protection system [known as *Seguro Popular] (SP)*, covering those under the informal sector or at the poverty level.

The four LAC countries implemented programs to expand the breadth of UHC and to address the needs of specific populations.

For instance, Argentina strengthened PHC by introducing preventive and chronic conditions health programs [23]. Brazil ESF expanded coverage of marginalized populations and improved health outcomes for people living in remote areas [15, 17].

The POS subsidized regime enrollment grew by 24% between 2000 and 2011, reaching more uninsured people in Colombia [24–26]. In addition, the 2015 “*Ley Estatutaria de Salud”* [Statutory Health Law] standardized coverage by the POS, and controlled health resources, aiming to limit the number of Tutelas [lawsuits] pursuing coverage for technologies not included in the POS [27].

Mexico created the *Seguro Medico para la Nueva Generation* [Health Insurance for the New Generation], providing comprehensive health coverage to all children born after December 2006 and those under-five-year-olds who lack health coverage [28]. In addition, *Catalogo Universal de Servicios de Salud* [Universal Catalog of Health Services] (CAUSES) expanded covered services to surgical procedures and included diseases with catastrophic expenditures [29].

Argentina and Brazil strengthened their human resources at the PHC level. The first implemented *Programa de Medicos Comunitarios* [Primary care doctors] (PMC) [20]; the second, voluntarily recruiting foreign physicians through the “*Mais Medicos*” [More Doctors] program, then signing an agreement with Cuba to supply these providers [30]. Unfortunately, PMC was discontinued in 2007 due to lack of funding; “*Mais Medicos*”, after years of strong criticism during Rousseff’s mandate, was terminated by the Bolsonaro administration [31].

### Access to medicines and health products

The healthcare system must guarantee the access and affordability of medicines, vaccines, and medical technologies (medical devices, diagnostics, blood and blood products) in a quality and timely manner, including policies to reduce the out-of-pocket expenditure on medicines.

All four of these countries expanded access to essential medicine, which, when paired with other interventions, strengthened service coverage [16, 19, 20, 32–34].

In 2002, Argentina launched *Programa Remediar* [Remedy Program] (REMEDIAR) incorporating the prescription of generic medicines targeting chronic conditions at PHC [16, 19, 32]. In 2008, REMEDIAR was integrated into the country health service networks (REMEDIAR + REDES) and expanded to all citizens nationwide [20].

The SUS in Brazil provides coverage to medicines through several programs that include a 20% expanded access to essential medicines [33]. Brazil offers an extensive free immunization program [16].

The coverage of medicines for chronic conditions in Colombia is entirely managed by the *Entidades Promotoras de Salud* [Health Promotion Organization] (EPS), although the POS lists the medicines that should be included in the national formularies.

Mexico’s *"SP"* provides access to low-cost medicines for chronic conditions, but on a smaller scale when compared to Argentina and Brazil programs. SP also provide limited access to certain high-cost treatments [14]. Vaccinations are covered for all Mexican children regardless of socioeconomic status or health insurance.

### Access to high-cost medicines and technologies

Although comprehensive oncology care and access to high-cost drugs are provided in the four countries, inequities still exist for people treated under the public healthcare system [18, 29, 32, 34]. The growing demand for new medicines and health products poses financial hurdles in these countries, and drug pricing constitutes a significant barrier to their access.

Brazil faced numerous lawsuits over the last 20 years, demanding the coverage of high-cost drugs to treat cancer and certain rare or low-prevalence diseases [33].

In Colombia, the Statutory Law mandated the POS to determine the essential drugs and health technologies and those that should be excluded from the list. Many of the high-cost drugs are now covered by POS, reducing but not eliminating the lawsuits.

Different mechanisms were in place to ensure that health expenditure on medicines and medical products add value. Most countries established health technology assessment (HTA) processes [35]. Also, price negotiation or price control implementation like the Mexican centralized purchasing helped maintain fair access to high-cost treatments and control excessive drug prices [36].

### Financing, governance, stewardship, and health information systems

Most of the health financing mechanisms across the four countries use a mix of taxes (federal and state) and cash transfers that vary according to the type of fiscal policies [14–16, 18, 19, 21, 25, 27, 29, 37–39]. Argentina has the highest health expenditures, as by 2015 spent 10.2% of its GDP on healthcare, yet only 3% on public health expenditure with a per-capita health expenditure of $1390; however there were similar to public health expenses in Mexico and Brazil (2.2–3.0% and 3.3–4.5%, respectively) [18, 19, 22, 29].

Across all four countries, health system governance decentralization included variations in their degrees of success.

In Argentina, *the health* system operates at three levels (federal, provincial, municipal); however, due to a lack of regulation policies, this process is not equitable in some provinces [40].

Each state and municipal health secretariat manages the SUS in Brazil, regulated by the MOH. Besides, in many municipalities where the public sector cannot fulfill people’s needs, arrangements with private institutions to improve access to PHC are in place [41].

Alike, in Mexico, since the health law reform, each state health secretariat administrates the SP throughout the *Regimenes Estatales de Protection social de Salud* [state regimens for social and health protection] (REPSS) [29, 38].

Law 100 in Colombia transferred operations to the municipalities establishing the managed competition among private organizations (EPS), which contract or own their *Instituciones Prestadoras de Salud* [Healthcare Providers] (IPS). Besides. in a country where health is considered an “economic asset”, this strategy of managed competition would be considered as an “open market initiative” rather than a PPP [26].

The PPP model in Brazil is highly recognized, especially for advanced medical practices (surgical oncology, cancer care, neurosurgery); Due to the long waiting list, the SUS subcontract medical organizations to manage these practices [22, 33]. However, significant controversy surrounding the recent introduction of foreign insurance companies and healthcare providers [22] to the market and the indiscriminate, not outcome-based payments for these organizations [39, 42].

The Mexican government has collaborated agreements with private providers to strengthen the quality of care provided through SP, ensuring delivery of cost-effective treatments for chronic conditions [43].

Brazil has long sought societal participation in policymaking on its federal, state and municipal health councils [44]. However, the lack of political will and policies to legitimize citizen involvement have contributed to structural and financial hurdles [17, 33]. This is similar in Colombia, while recent passage of Statutory Law promoted increased societal participation, many decision-makers perceive citizens as being ill-equipped for these deliberative processes given their lack understanding of health as a public good [45].

## Discussion

Although numerous publications analyze both the evolution of healthcare systems and adoption of UHC across Latin America, this review utilizes elements from the WHO Regional monitoring frameworks and the 13th program of work to examine how UHC has been incorporated into regional and country-level health systems.

Each of the four countries have strengthened their health services coverage by establishing patient-oriented health systems that expand access to health services, especially PHC, increasing the perceived quality of care [3, 29, 32]. The LACs developed numerous country-specific measures to develop UHC through either expanded coverage of health services or through strengthened financial protections.

- Argentina built the foundation of UHC by a strong improvement of PHC model and the inception of national programs to control NCDs supplemented by REMEDIAR program. This allowed to achieve “nominal” UHC, meaning that people enrolled in the healthcare systems have the right to access them [18].
- In the last 10 years, Brazil expanded coverage to 62% of citizens by quadrupling the number of people covered by the ESF program [17]. Also strengthened the access to medicines and health products and incorporated the participation of the private sector to reduce waiting times to access certain medical procedures.
- Since the inception of Statutory Law, Colombia uniformed POS components, excluded obsolete technologies from POS, increased the funding through UPC and limited the number of Lawsuits *[Tutelas]*.
- The establishment of SP and SMNG in 2004 allowed to mitigate asymmetries for those without formal coverage. In 2012, Mexico claimed the achievement of UHC (standing as another example of “nominal” UHC) [27]. Then, as part of the 2014 SP health law reform, a 13-fold increase in Federal investment from 11 million (2004) to 146 million Mexican pesos (2013) was observed [14]. The decentralization of the *Comision Nacional de Proteccion Social en Salud* [National Commission of Social Protection in Health] and the REPSS, two pooling mechanisms, allowed to a 9% decrease (from 52.2 to 41.4%) in out-of-pocket health expenditures, and a 43% increase in coverage, adding 53.5 million people in 2018 [14, 29].

By contrast, these UHC initiatives have been faced with challenges surrounding the lack of strong financial protection measures a which continue to put many people at risk of catastrophic health expenditures. There is still debate surrounding the ideal financing mechanisms for UHC in low to middle-income countries (LMICs), and no clear guide regarding tax funding apart from the recommendation to allocate 5% of the GDP posing challenges stemming from uneven tax collections, and the increase in out-of-pocket expenditure [12].

According to WHO and WB data, in Argentina 5% of the population spent more than 25% of household expenditures in healthcare, while in Brazil 3% is noted [46].

- Argentina metrics showed that even with strong financial incentives and an increase in the pooling of funds, unmet healthcare needs persist, mainly in provinces that suffer a lack of health providers, outdated health information systems, and low institutional capacity. The fragmentation in healthcare funding has led to ineffective funding policies at both the state and sub-national level. Most of these health coverage initiatives rely on the external funding, meaning these are controlled by the MOH or the Ministry of Finance rather than each Argentinian province [18, 20, 41]. Health governance issues must be resolved in order to ensure access to quality health systems; financial readjustments are insufficient, as emphasized by Uribe-Gomez [27].
- Brazil’s SUS continues to be underfunded as federal health funding stagnate with public health spending increasing only 3.2% over the last 10 years [21]. SUS receives only 46% of the available funds slated for public health [42]. This has led to an increase in patient cost-sharing (e.g., out-of-pocket expenditures) for persons in the lower economic strata, as well as nearly 400,000 lawsuits related to insufficient health coverage [47]. Two studies found that out-of-pocket health spending was higher in the groups covered by this program, even when compared to higher-income populations with private medical insurance [47]. Recent models of the Brazilian health system have shown that the country must increase its annual contribution by the central government to the municipalities by 3%, to guarantee and maintain UHC targets, such as infant mortality and access to ESF [48].
- Colombia has also been plagued by underfunding of its subsidized POS component, as well as high rates of informal workers evading their health contributions. Insufficient funding has furthered health inequities [24]. This research has shown that, under these conditions, even with FOSYGA cross-subsidization it does not guarantee the adequate funding [27].
- Mexico faced a 25% deficit in health spending and appropriate funding for SP would require resource pooling and cooperation between the states and central government [14]. During 2019, Mexican President Lopez Obrador stressed the need of SP transformation, resembling the *Instituto Mexicano de Seguros Sociales* [the Social Security Institute], the largest Mexican social security institution [49, 50].

UHC remains an important policy agenda for many countries, including the four LACs, where achieving UHC requires more than health system reforms and financial protection [8, 51, 52].

Argentina is thriving towards more effective UHC, especially by introducing capitated payments transferred to the provinces, which enable coverage for services included in their UHC health benefits packages [3, 18]. Since 2020 Mexican *Instituto de Salud para el Bienestar* [Institute of Social Welfare], provides comprehensive health coverage replacing SP, having its structure and governance mechanism funded by central mechanisms.

Many LAC continue to develop new initiatives in their quest for UHC. Prioritized health services baskets (benefits packages) select healthcare interventions that demonstrate cost-effectiveness and might improve the UHC index by expanding access to high-value health services, while reducing the patient’s financial burden. This methodology could be applied to prioritize essential services for other countries, as the *Regimen de Garantias Esplicitas en Salud program* [Regime of Specific Guarantees in Health] (AUGE), in Chile did, and as it has been done in other LMICs [5, 53, 54].

Chile’s AUGE offers both an extended benefits package and financial protection measures funded mainly through VAT taxes [55]. Moreover, in 2015, the Senate approved Ricarte Sotto bill, which created a fund to guarantee comprehensive care, including drugs, devices, and procedures for certain diseases not included in AUGE catalog.

These could be achieved by improving tax policies, such as including a mandatory contribution (i.e. for catastrophic expenditure) or by involving the private sector to support structural inefficiencies in healthcare, or through the provision of funds by external donors, if required.

Finally, strengthening PHC is a turnaround in health systems to commit to UHC and SDGs, as well as to include challenges posed by non-communicable diseases (cardiovascular disease, diabetes), as well as injuries, and emerging diseases with pandemic potential [3, 5, 51, 56]. This is an example of Argentina, that is expanding their PHC network at a wider scale allowing to maintain a continuum between the different levels of care.

In keeping with UHC aims, these following measures could be considered to enhance the health service delivery, while minimizing financial risk to patients

- First, decentralize the PHC and transition their management to each region (community, municipality). To guarantee access to specialized healthcare services (i.e. surgical services or diagnostic tests) while avoiding excessive wait times, a referral program between the PHC and specialty care, usually run by the MOH, should be available. Co-payments for the use of these centers need to be avoided.
- Second, find an efficient mechanism for the healthcare system financing, especially for the primary care. Channeling of funds from the central government to autonomous regions, conditional cash transfers, and implementation of “progressive” mechanisms for health expenditure have all been shown to be an efficient way to finance the PHC [18]. However, as the PAHO recommends allocating 30% of healthcare expenditure to PHC, coordination between finance and health authorities is required since political and economic instability faced by many countries in LAC might challenge the implementation of this recommendation [57].
- Third, involving private organizations could be considered in countries with healthcare system structural or technical issues that may conflict the UHC. In the case of Southern Africa where private organizations were contracted to deliver PHC [58], or in Brazil where established agreements with NGOs to provide human resources for the first level of care, are such examples [39]. This requires that MOH controls the private sector performance with an outcomes-based approach. Healthcare governance with clear roles and responsibilities is required to guarantee that healthcare is being delivered equitably.

The information covered in this report supports the assessment, planning, and execution of those measures that enhance both service provision and financial protection mechanisms in an aim to improve access to comprehensive care, reflecting the UHC purpose.

Finally, we carried out this research before the Coronavirus Disease 2019 (COVID-19) pandemic. As a public health emergency, COVID-19 challenged healthcare systems globally, either by increasing pressure in healthcare facilities, demonstrating the long-standing shortage of trained human resources for health, and recently with delaying access to vaccines in low-income countries. The LAC countries presented challenges in the access to healthcare facilities, for example, the lack of tertiary centers, as noted by the pressure in Brazilian hospitals, which saturated the PHC and raised the fatalities n rural communities, and the lack of trained healthcare providers in Argentinean intensive care units.

By 2021 the new Coronavirus strains, and the delay in vaccine delivery, will continue to put hurdles in these countries. It would be interesting to analyze equitable access to universal healthcare in the context of a pandemic.

We must mention several limitations in this review:

Despite that Argentina, Colombia, Brazil, and Mexico were the countries of interest in this research; other countries that moved towards UHC (i.e., Costa Rica, Panama, Chile) are missing. Secondly, we analyzed and described UHC coverage index elements from an established framework, but little evidence assessing equitable access to healthcare, vaccination policies, and coverage to vulnerable populations (elderly, people at poverty levels, or living in isolated rural areas and sexual minorities) were found. We acknowledge that missing these issues could provide a blind and not generalizable point of view of the current situation in the region.

Semi-structured self-surveys are a direct way to collect information easily but are plagued by low response rates [59]. Our research aimed to survey at least 12 stakeholders across the countries of analysis. However, due to the extensive nature of these surveys, only three stakeholders per country were included in this analysis, making the data analysis more descriptive rather than quantitative. An alternative to collect data more directly would have been to interview these stakeholders by phone. We consider that the development of UHC in LAC could provide more food for thought and would require further research, mainly involving the stakeholder’s perception through traditional qualitative methods.

## Conclusions

The four countries have made progress in the service coverage by implementing primary care reforms, and by incorporating of certain elements into their national health programs, such as subsidized essential medicines. However, these countries lag in providing strong financial protections from high medical bills based on WHO’s global figures. While no country has achieved true UHC for all its citizens, UN member countries must develop the capabilities and strategies to achieve UHC if they are to meet WHO’s goal of covering two billion people by 2030.

Future targets for health system development in LAC include developing a sustainable PHC network (integrated health services networks) that would be capable of reaching more than 85% of the population at need [57]. Comprehensive health services includes family physicians and community healthcare workers as cornerstones of care, complemented with the provision of essential medicines, and access to childhood immunization programs. Non-communicable diseases prevention programs and the enhancement of social protection mechanisms are examples of strengthening PHC while providing financial protection. Last by not least, a redesign in healthcare networks to be capable to cope with future pandemics.

## Supplementary Information

**Additional file 1.**

## Data Availability

“Not Applicable”.

## Abbreviations

AUGE: Acceso Universal con Garantias Explicitas (Chile)
CNPSS: Comision Nacional de Proteccion Social en Salud (Mexico)
EPS: Empresa Prestadora de Salud (Colombia)
ESF: Estrategia Saúde Familiar (Brazil)
FOSYGA: Fondo Solidario y de Garantias (Colombia)
GDP: Gross domestic product
HIS: Health information systems
HIV/AIDS: Human immunodeficiency virus
IPS: Instituciones Prestadoras de Salud (Colombia)
ISB: Instituto de salud para el bienestar (Mexico)
LAC: Latin America and the Caribbean
LMICs: Low to middle-income countries
LSHTM: London School of Hygiene and Tropical Medicine
MoH: Ministry of Health
NCD: Non-communicable diseases
NGOs: Non-Government Organizations
PAHO: Pan American Health Organization
PCCs: Primary care centers
PHC: Primary health care
POS: Plan Obligatorio de Salud (Colombia)
PPP: Public-private partnerships
REPS: Regimenes de proteccion en salud (Mexico)
SDG: Sustainable Development Goals
SESA: Servicios Estatales en Salud (Mexico)
SHS: Suplementar Health Subsector (Brazil)
SMNG: Seguro Medico para la Nueva Generacion (Mexico)
SP: Seguro Popular (Mexico)
SUS: Sistema Único de Saúde (BRAZIL)
UHC: Universal health coverage
WHO: The World Health organization
