# Supplementary material for: Has Latin America achieved universal health coverage yet? Lessons from four countries
Source: Arch Public Health. 2022 Jan 21;80:38. doi: 10.1186/s13690-022-00793-7 (PMC8777418; doi:10.1186/s13690-022-00793-7)
Supplement: Supplementary file 1 — Additional file 1. [file 13690_2022_793_MOESM1_ESM.docx]

SUPPLEMENTARY MATERIAL – (APPENDIX)

**Search strategy:**

**Appendix 1:**

PubMed

(Universal Health Insurance[Mesh] OR Universal Cover*[tiab] OR Universal Health*[tiab] OR UHC[tiab] OR Universal Insurance[tiab]) AND (Argentina[Mesh] OR Argentin*[tiab] OR Brazil[Mesh] OR Brazil*[tiab] OR Mexico[Mesh] OR Mexic*[tiab] OR Colombia[Mesh] OR Colombia*[tiab])

**LILACS**

(MH Universal Health Insurance OR ((Cover$ OR Cobertura OR Insurance OR Seguro OR Healthcare OR Salud OR Saude) AND (Universal))) AND (MH Argentina OR Argentin$ OR MH Brazil OR Brazil$ OR Brasil$ OR MH Mexico OR Mexic$ OR OR MH Colombia OR Colombia$)

**Appendix 2 -**

**Table – Country Comparison with findings from the Scoping review**.

Source: Own elaboration – References are inserted in the manuscript.

|  |  | Argentina | Brazil | Colombia | Mexico |
| --- | --- | --- | --- | --- | --- |
| Healthcare service delivery | Expansion of UHC/PHC | *Introduction of Plan Federal de Salud (2004) as the cornerstone of UHC based on PHC* | SUS establishment based on strong PHC model. | Law 100 (1993) reformed established the Mandatory Health Benefit Package (POS) under two regimes, the contributive and the subsidized (for those without formal employment) | *Establishment of Seguro Popular (2004)  "Seguro Medico para la nueva generacion” (2007) improved coverage for childrens* |
|  | Implementation preventive health initiatives and early detection programs | *Plan Nacer / SUMAR:* PHC oriented maternal and child health programs; Plan *REDES* expanded to provide coverage for all ages at PHC *)* [19,20] Expanded disease management programs including sexually transmitted infections, cardiovascular disease, diabetes, and other chronic conditions. [18,23] | *ESF* introduced to increase coverage in underserved areas and reduced mortality from both non-communicable and infectious diseases, especially infant mortality due to diarrhea and respiratory infections. Improvement of pre-natal care and sexual and reproductive health programs. [15–17,21,40] | 2015 *Statutory Law* (*Statutury Law*) tend to strengthen and unify POS (based on World Bank UHC model), increasing its breadth and reducing lasymetries among the two groups [27] | I  mprovement in the integral coverage of hypertension, diabetes, included 1,807 ICD 10 diagnostic categories, 294 surgical procedures, and treatments for 65 different catastrophic diseases.Also birth control and integral pregnancy management for 90% of the population under Seguro Popular. |
|  | Human resurces for PHC | Programa de Medicos Comunitarios applied differential payments to family medicine and general practice physicians working within PHC centers*^.^* [23] | “Mais Medicos” program recruited 1500 foreign physicians to cope with shortage of PHC level. Then Cuban and Brazil's Government agreed that the first will provide primary care physicians to increase the breadth of healthcare delivery [59] |  |  |
| Access to Medicines and Health Products | Provision of Essential Medicines and vaccines | *REMEDIAR* provided pharmaceutical assistance at PHC level covering 85% of drugs for Diabetes, Hypertension, chronic pulmonary disease among other chronic conditions [19,20] | *Farmacia Popular* Do Brasill, *Dose Certa, Remedio em Casa* programs subsidized costs in treatments for diabetes, hypertension, asthma, and cardiovascular diseases. *National Immunization program providing free vaccination.*  one et al., 2017; Macinko and Harris, 2015; Muzaka, 2017) | Medicines and health products are included in the POS lists managed by the EPS; Before the POS unification (2008) and the Statutuory Law, large proportion in out of pocket expenditure on essential medicines were seen. | Subsidization for medicines to control chronic diseases.  Vaccination cards are available for Mexican Children’s independently of socioeconomic status and health insurance |
|  | Extended coverage for diseases with social impact or limiting chronic conditions | Comprehensive care programs for HIV and cancer guarantee treatments for patients w/o coverage [19,20]  Colorectal Cancer, Cervical and Uterine Cancer, Diabetes, Smoke cessation programs provide free of charge treatments [18,23] | Other diseases like nutritional and bleeding disorders, cholera, Chagas disease, dengue, schistosomiasis, filariasis, leprosy, HIV/AIDS, influenza, leishmaniasis, malaria, meningitis, systemic mycoses, multiple myeloma, human rabies, trachoma, and tuberculosis have expanded coverage. | Statutory *law* mandated the creating of a list of medicines and health products that should be removed from POS due to the lack of clinical benefit and value for the people. *After the POS negative list creation, all medicines included are funded by the Capitation Payment Unit (UPC).* |  |
|  | Implementation of Health Technology Assessment or value demostration for high cost drugs | UCEETS Office established in 2009 to control quality of care and assess health technologies. In 2018 the National Commission of Health Technology Assessment (CONETEC) was created under the MOH umbrella, as the first multistakeholder governing body for HTA. [35] | In 2011 of Comissão Nacional de Incorporação de Technologias No Sistema Único de Saúde [National Commission for the Incorporation of Technologies in the Unified Health System] or CONITEC was stablished to determine the value of technologies funded by SUS, including horizon scanning to identify emerging technologies and anticipate the effects such technologies may have on the health system. | In 2011 The Instituto de Evaluaciones Tecnologicas en Salud (Institute for Health Technology Assessment) or IETS was created, to inform drug coverage for decision makers or defining eligible populations, dose or treatment duration, or line of therapy through clinical guidelines. [35] In 2017, resolution 330 established the procedure involving different stakeholders (including the public), to decide which technologies should be disinvested. Then, the MOH underwent a deliberative process to prioritize and create a negative POS list [27] | In 2004 Mexican Centro Nacional de Excelencia Tecnologica en Salud [National Center for Health Technology excellence – CENETEC] was stablished to create clinical guideliness and assess the value of health technologies. Then, the General Health Council define listing/inclusion of drugs and devices in the national formulary (called Cuadro Basico).  Government established centralized purchasing after the inclusion in the national formularies. The Coordinating Commission for the Negotiation of Prices of Medicines and Health Supplies) negotiates with the manufacturers the price of the drugs included on its “essential medicine list” considering “value for money” or budget impact with pricing regulations based on reference pricing at the national level [35] |
|  |  |  |  |  |  |
| Financing, Governance, Stewardship and Health Information Systems | Healthcare Financing (Taxation) | Value added Taxes collected by the Ministry of Financce. Provincial and municipal taxes collected and managed independently. [20] | Central Tax revenue; Budgets from statal and municipal governments, are the SUS source of funding. (17) | Government contributions and municipal taxes, including gambling taxes.  Law 100 established the UPC a fixed amount that government pay to EPS per insured per fiscal year. [27] | The Mexican SP is highly-subsidized through federal and state taxes, with the federal government contributing 83% of annual per-person cost and states funding the balance) |
|  | Sustainable financing mechanisms (Cross-funding, etc) | Central government transferred more than USD 234 million to provinces to fund primary health strategy over *SUMAR* and provide financial aid programs, such as Asignación Universal por Hijo [Universal Child Allowance] and Asignación Universal por Nacimiento [Universal Birth Allowance] [20] | Bolsa de Família [Family Grant], unifies multiple social initiatives to provide extra cash to the poorest, conditional upon school attendance and use of PHC services. Strong criticisms about underfunding of SUS [42] Federal spending on healthcare unchanged over 15 year period, slight increase of 0,7% GDP in public health expenditure over 5 year period [21] | Labor contributions are based on fixed proportion of the income collected by the government under FOSYGA; 1.5% of funds from payroll taxes were transferred from the contributive regime to the subsidized POS as a cross-subsidization *)*[24] | Descentralization of CNPSS and REPSS decreased out of pocket health expenditure and increased number of people under SP. ( |
|  | Descentralization of healthcare provision mechanisms and PPP | The country lack of decentralization policies that regulate health provision from central to subnational levels with lower primary care orientation in the provinces due to poor institutional capacities [20] | Decentralization of healthcare services and budget management responsibility at municipal level. [15] NGOs were contracted to to provide support to public health services [39] |  | Implementation of PPP to increase coverage to NCD has been cost-effective. |
|  | Advances in HIS (including Electronic health records) | HIS is still in development and the country lacks of connectivity across the entire health [[20] | Lower development of unified HIS in the public healthcare services [17] |  | SP move towards accreditation of facilities, investments in technology and qualified human resources. |
